# Altered Gut Microbiota in Korean Children with Autism Spectrum Disorders

**DOI:** 10.3390/nu13103300

**Published:** 2021-09-22

**Authors:** Sungji Ha, Donghun Oh, Sunghee Lee, Jaewan Park, Jaeun Ahn, Sungku Choi, Keun-Ah Cheon

**Affiliations:** 1 Department of Psychiatry, Institute of Behavioral Science in Medicine, Yonsei University College of Medicine, Seoul 03722, Korea; neuroscience79@gmail.com; 2 Graduate School of Medicine, Yonsei University College of Medicine, Seoul 03722, Korea; dhun0329@gmail.com; 3 Research Lab., Ildong Pharmaceutical Co., Ltd., Hwaseong 18449, Korea; slee@ildong.com (S.L.); pjw250@ildong.com (J.P.); alanchoi@ildong.com (S.C.); 4 Division of Child and Adolescent Psychiatry, Department of Psychiatry, Severance Children’s Hospital, Institute of Behavioral Science in Medicine, Yonsei University College of Medicine, Seoul 03722, Korea; jaeun.ahn87@gmail.com

**Keywords:** autism spectrum disorder, microbiome, microbiota-gut-brain axis, Korean

## Abstract

Autism spectrum disorder (ASD) is a neurodevelopmental disorder characterized by social and behavioral impairments. Recent studies have suggested that gut microbiota play a critical role in ASD pathogenesis. Herein, we investigated the fecal microflora of Korean ASD children to determine gut microbiota profiles associated with ASD. Specifically, fecal samples were obtained from 54 children with ASD and 38 age-matched children exhibiting typical development. Systematic bioinformatic analysis revealed that the composition of gut microbiota differed between ASD and typically developing children (TDC). Moreover, the total amounts of short-chain fatty acids, metabolites produced by bacteria, were increased in ASD children. At the phylum level, we found a significant decrease in the relative Bacteroidetes abundance of the ASD group, whereas Actinobacteria abundance was significantly increased. Furthermore, we found significantly lower *Bacteroides* levels and higher *Bifidobacterium* levels in the ASD group than in the TDC group at the genus level. Functional analysis of the microbiota in ASD children predicted that several pathways, including genetic information processing and amino acid metabolism, can be associated with ASD pathogenesis. Although more research is needed to determine whether the differences between ASD and TDC are actually related to ASD pathogenesis, these results provide further evidence of altered gut microbiota in children with ASD, possibly providing new perspectives on the diagnosis and therapeutic approaches for ASD patients.

## 1. Introduction

Autism spectrum disorder (ASD) is a neurodevelopmental disorder characterized by persistent social communication deficits, with restricted and repetitive patterns of behaviors, interests, or activities [1]. According to a recent report, ASD prevalence in 2016 was reported to occur in 18.5 per 1000 children aged 8 years in the United States [2]. In particular, ASD prevalence in South Korea was estimated to be 2.64%, which had been attracting worldwide attention [3]. As ASD prevalence increases over time, the social burden also increases significantly. In fact, the cost of supporting an individual with ASD in a lifespan was approximately $1.5–2.4 million in the United States and £0.92–1.5 million in the United Kingdom [4].

Although the cause of ASD has yet to be identified, recent studies have reported that the gut microbiome can play a role in its pathogenesis [5]. Particularly, changes in the intestinal environment caused by the gut microbiota have been found to affect the production of signaling substances, consequently affecting mature brain functioning as well as prenatal and postnatal central nervous system (CNS) development [6]. This connection, which is called the microbiota–gut–brain axis, refers to the bidirectional communication pathway between gut bacteria and the CNS and is known to be associated with various processes, including neuroinflammation, stress axis activation, neurotransmission, blood-brain-barrier (BBB) formation, myelination, microglia maturation, and neurotransmitter synthesis [7,8]. As an example, several researchers have confirmed that germ-free mice exhibited increased hippocampal neurogenesis, increased BBB permeability, and abnormal behaviors, such as decreased sociality and increased locomotor activity [9,10,11,12], showing that normal gut microbiota can modulate brain function and behavioral outcomes. Furthermore, Sharon et al. confirmed that microbiota colonization from ASD patients was sufficient to induce autistic behaviors in mice, and microbial metabolites were found to improve abnormal behaviors in an ASD mouse model [13].

Gastrointestinal (GI) problems, including abdominal pain, constipation, and diarrhea, are commonly observed in ASD patients [14,15,16]. Additionally, behavioral problems in ASD patients were related to GI disturbance severity, wherein ASD patients with GI disturbances showed lower social skills, higher anxiety, and more frequent aggressive behaviors than those without GI issues [17,18]. These associations between ASD and GI problems suggest a possible significant relationship between the gut microbiota and ASD.

Notably, several case-control studies have affirmed the possible roles of gut microbiota in ASD pathogenesis through the analysis of aberrant gut microbiota compositions in ASD patients. The most common findings were a decreasing trend in the Bacteroidetes to Firmicutes ratio [19,20,21] and a higher *Clostridium* abundance in ASD patients than in normal controls [22,23,24]. Despite these findings, there is little consensus on the phenotypic signature of the gut microbiome in ASD patients. One of the reasons for these inconsistent results is that the microbiome composition can be influenced by various factors, such as diet, lifestyle, and medical history [5]. In fact, it has been shown that the gut microbiota can be stably altered by dietary changes and exposure to xenobiotics, including antibiotics [25].

Although the role of the microbiome in ASD pathogenesis has not been identified, many interventional studies based on microbiome modulation have been conducted. Liu et al., for one, showed that the probiotic *Lactobacillus plantarum* PS128 (PS128) had beneficial effects on the opposition/defiance behaviors of boys with ASD [26]. Kang et al. also found that the microbiota transfer therapy improved GI and behavioral symptoms in ASD children, additionally confirming that these effects persisted two years later [27,28]. At present, these results should be interpreted carefully, but it is expected that microbiome-based interventions can be an alternative to help children with ASD in the future. Therefore, various basic studies reflecting each race and culture are required. 

In the present study, we aimed to identify gut microbiota profiles associated with ASD through the intestinal microflora analysis of Korean children with ASD and children with typical development. Specifically, the fecal microbiota of each participant was analyzed through the V3–V4 regions of the 16S ribosomal RNA (rRNA) sequencing, and the main microbiome metabolites and short-chain fatty acids (SCFAs) were analyzed to understand the microbiota–gut–brain crosstalk.

## 2. Materials and Methods

### 2.1. Participants and Study Design

This study was approved by the Institutional Review Board of the Severance Hospital, Yonsei University College of Medicine (4-2018-0745). Sample collection began in October 2018, wherein participants who visited the Severance Children’s Hospital agreed to serve as fecal donors, providing written informed consent and questionnaire data sheets. Specifically, fecal samples were collected from 54 ASD children (4–13 years of age; 43 male and 11 females) and 39 typically developing children (TDC) (4–9 years of age; 18 males and 21 females). One sample of a girl from the TDC group was excluded from the data analysis since the extracted DNA did not pass the quality control. ASD participants were diagnosed by a child and adolescent psychiatrist based on the Diagnostic and Statistical Manual of Mental Disorders, 5th Edition [1], which was supplemented by the Autism Diagnostic Observation Schedule-2 (ADOS-2), Autism Diagnostic Interview-Revised (ADI-R), and Social Responsiveness Scale (SRS) [29,30,31]. Meanwhile, participants in the TDC group were screened by a child and adolescent psychiatrist based on clinical examinations, including intelligence tests, SRS, and direct patient interviews. All participants were also restricted from taking antibiotics for 3 months and probiotic supplements for 15 days prior to fecal sample preparation.

### 2.2. Sample Collection and DNA Extraction

Fecal samples were individually collected by parents at each home using an in-house collection kit, which were immediately stored at 4 °C and transferred to the laboratory within 20 h. Fecal samples (100 mg, wet weight) were then mixed with 200 μL of phosphate-buffered saline (PBS; pH 7.4; 1:9; PBS: saline), and DNA was extracted using the QIAamp DNA Mini QIAcube Kit (#51326, Qiagen, Hilden, Germany). The DNA concentration and quality were determined using 1% agarose gel electrophoresis and NanoDrop ND-1000 spectrophotometry (NanoDrop Technologies Inc., Wilmington, DE, USA). Afterwards, genomic DNA was stored at −80 °C before delivery to a commercial sequencing facility (Macrogen, Seoul, Korea).

### 2.3. Next Generation Sequencing Processing

Genomic DNA was amplified by polymerase chain reaction (PCR), which used a universal primer set (341F and 805R) to target the V3-V4 region of the 16S rRNA gene, also allowing the Illumina overhang adaptor, where the forward primer (341F, 5′-TCGTCGGCAGCGTCAGATGTGTATAAGAGACAGCCTACGGGNGGCWGCAG-3′; the underlined sequence indicates the target region primer) and the reverse primer (805R, 5′-GTCTCGTGGGCTCGGAGATGTGTATAAGAGACAGGACTACHVGGGTATCTAATCC-3′) are found, to be used [32]. DNA amplification was performed under the following conditions: initial denaturation at 95 °C for 3 min, followed by 25 cycles of denaturation at 95 °C for 30 s, primer annealing at 55 °C for 30 s, and extension at 72 °C for 30 s, with a final elongation at 72 °C for 5 min. Secondary amplification for Illumina NexTera barcode attachment was then performed with the i5 forward primer (5′-AATGATACGGCGACCACCGAGATCTACAC-XXXXXXXX-TCGTCGGCAGCGTC-3′; X indicates the barcode region) and i7 reverse primer (5′-CAAGCA GAAGACGGCATACGAGAT-XXXXXXXX-GTCTCGTGGGCTCGG-3′). The amplification conditions were similar to those described above, with the exception of the amplification cycle, which was set to eight. PCR products were confirmed by 1% agarose gel electrophoresis and visualized using a Gel Doc system (BioRad, Hercules, CA, USA) afterwards.

Amplified products were purified using a CleanPCR kit (CleanNAc, Inc., Waddinxveen, The Netherlands), wherein equal concentrations of purified products were pooled, and short fragments (non-target products) were removed using CleanPCR (CleanNA, Inc., Waddinxveen, The Netherlands). DNA quality and product size were then assessed using a Bioanalyzer 2100 (Agilent, Palo Alto, CA, USA) with a DNA 7500 chip. Afterwards, mixed amplicons were pooled, and sequencing was carried out at Macrogen, Inc. (Seoul, Korea) using the Illumina MiSeq Sequencing system (Illumina, San Diego, CA, USA) following the manufacturer’s instructions.

The raw Illumina read data for all samples were deposited in the European Bioinformatics Institute European Nucleotide Archive database under the accession number PRJEB45948.

### 2.4. Bioinformatic Processing

We obtained demultiplexed, paired-end reads from the MiSeq platform and imported them into the Quantitative Insights into Microbial Ecology 2 (QIIME2, ver. 2020.2.0) software pipeline using the FASTQ manifest protocol [33]. Primers in the raw sequences were trimmed using the Cutadapt software [34], and paired-end reads were merged using the VSEARCH merge pair plugin [35]. The resulting merged reads were then filtered to exclude low-quality reads based on a minimum quality score of q30, removing ambiguous base calls and all chimeric sequences by applying the vsearch uchime_ref plugin [36]. Following this, multiple high-quality sequence alignments were performed using MAFFT ver. 7, a method suitable for multiple alignments of a large number of short sequences [37]. Uninformative base positions derived from the lane mask were removed, and the resulting aligned sequences were used to generate a phylogenetic tree using FastTree 2.1.10 [38]. Additionally, the EzBioCloud database, which is complementary to QIIME 2, was used for taxonomic analysis based on 80% identity using the BLAST+ consensus taxonomy classifier [39,40].

Afterwards, alpha and beta diversity metrics were extracted using the core-metrics-phylogenetic plugin, which were based on the phylogenetic and non-phylogenetic trees. Species richness and evenness were then compared based on the Faith-phylogenetic diversity and operational taxonomic units (OTUs) and the Shannon index and pielou_e, respectively [41,42]. Lastly, the overall phylogenetic distance between the two groups was estimated using weighted UniFrac dissimilarity based on the phylogenetic tree [43].

### 2.5. SCFA Analysis

One gram of feces was dissolved in 8 mL of distilled water and homogenized using a vortex mixer for 5 min. The solution was then centrifuged at 3273 *g* for 10 min at 25 °C. The resulting supernatant was filtered through a 0.2-μm cellulose acetate/surfactant-free membrane filter, and 20 μL was injected into a Waters e2695 HPLC system equipped with a Waters 2489 UV detector. Afterwards, chromatographic separation was conducted under isocratic elution conditions using a Concise coregel 87H3 column (7.8 × 300 mm, 9 μm) (Concise Separations, San Jose, CA, USA) and a mobile phase of 0.01 N sulfuric acid, with the detection wavelength set at 210 nm. Other chromatographic conditions included a column oven temperature of 35 °C, a flow rate of 0.6 mL/min, and a run time of 65 min. Following chromatographic separation, individual SCFAs (acetic acid, propionic acid, and butyric acid) were identified and quantified based on the known standard retention times and peak areas (HPLC grade, Sigma-Aldrich, St. Louis, MO, USA).

### 2.6. Statistical Analysis

The Kruskal–Wallis test with Tukey’s multiple comparisons was used to determine significant bacterial variables among the groups with ASD and TDC in phylum and genus levels. Additionally, this test was used for ratio analysis of Bacteroidetes to Firmicutes. Linear discriminant analysis effect size (LEfSe) was used to explore the potential bacterial biomarkers associated with different groups [44]. This algorithm was used not only for bacterial biomarker discovery but also for determining the functional differences between ASD children and TDC.

Alpha diversity indices, including the Faith phylogenetic diversity, OTUs, Shannon index, and pielou_e, were calculated using the QIIME 2 plugin, and the Wilcoxon test was used to estimate alpha diversity differences between each category using R version 4.0.2. Spearman’s rank tests calculated the correlation coefficients between SCFA and microbial richness, including Faith_pd and observed OTUs. For beta diversity, the significant distance difference among groups was assessed using permutation-based multivariate analysis of variance (PERMANOVA) with 10,000 replicates, which was plugged into QIIME 2. We analyzed the correlation between the relative bacterial abundances and Principal component 1 (PC 1) of the weighted UniFrac distance using Spearman correlation in R version 4.0.2. to determine the bacteria that have the most influence on the community structure. 

Based on the marker gene data and the reference genome database, the functional composition of the metagenome was predicted using phylogenetic investigation of communities by reconstruction of unobserved states (PICRUSts). The sequences were aligned against Greengenes ver.13.5., and Operational Taxonomic Units (OTUs) were assigned at 97% identity. The OTU table was uploaded to normalize by copy number, followed by metagenome prediction, and finally categorized by KEGG function on the Galaxy interface (http://huttenhower.sph.harvard.edu/galaxy, Galaxy Version 1.0.0). All descriptive statistics were processed using R version 4.0.2.

## 3. Results

### 3.1. Demographic Characteristics

In this study, we analyzed fecal samples from a total of 92 participants, including 54 ASD children and 38 TDC (Table 1). No statistically significant differences were observed in the mean ages of the two groups. However, the gender ratio, IQ, and SRS scores showed a significant difference between ASD and TDC groups (Table 1).

### 3.2. Microbial Profiling of the ASD and TDC

**Figure 1:**
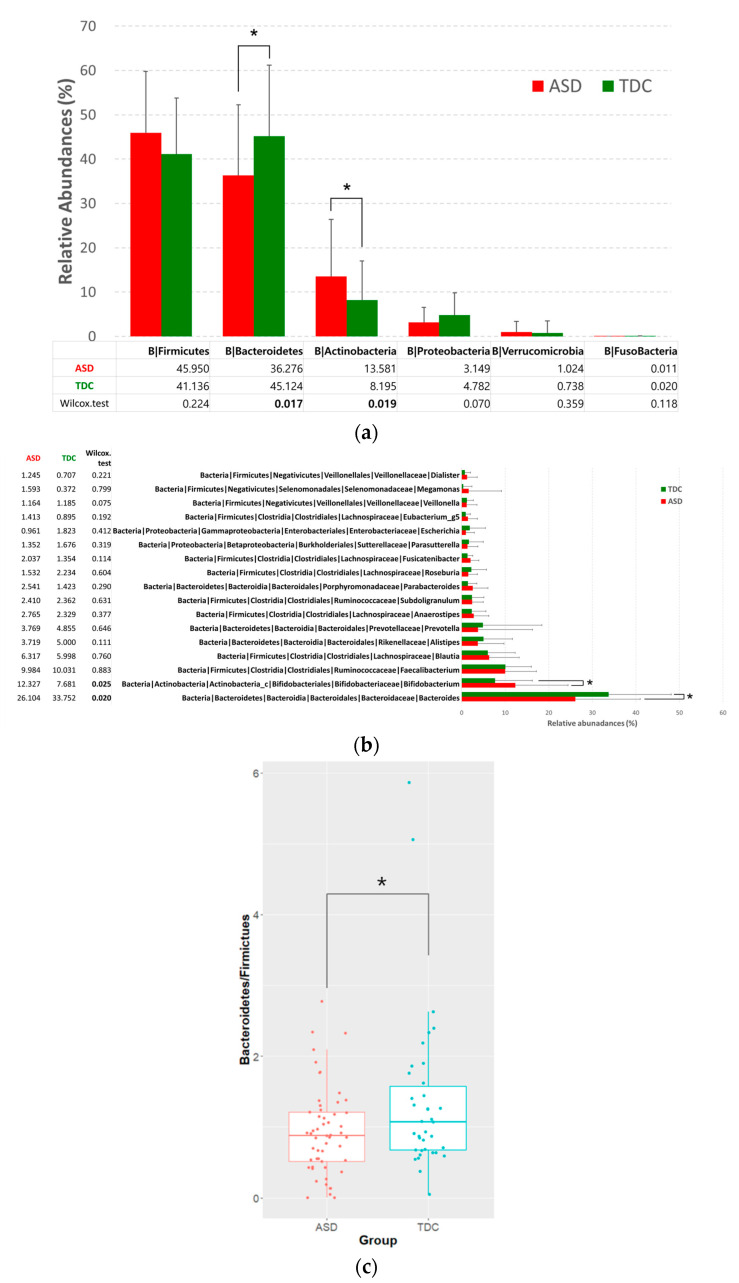
Microbial profiles at (**a**) the phylum and (**b**) the genus level and (**c**) Bacteroidetes/Firmicutes (B/F) ratio. The proportion of the bacterial composition is presented in tables (**a**,**b**). Error bars indicated standard deviation (S.D). Asterisk indicates significance between groups (* *p* < 0.05).

We performed a microbial taxonomic analysis to compare the ASD and TDC groups. We found significant differences in the gut Bacteroidetes and Actinobacteria proportions, which are representative bacterial phyla, between the two groups. Specifically, significantly higher Actinobacteria and significantly lower Bacteroidetes levels were observed in the ASD group than in the TDC group (*p* < 0.05) (Figure 1a). At the genus level, increased relative *Bifidobacterium* and decreased relative *Bacteroides* abundances were also observed in the ASD group as compared to that in the TDC group (*p* < 0.05) (Figure 1b). Furthermore, the Bacteroidetes to Firmicutes ratio was found to be significantly decreased in the ASD group (*p* < 0.05) (Figure 1c). Although no statistically significant differences were found at the species level, a decreasing tendency in *Bacteroides vulgatus* and *Bacteroides dorei* was also observed in the ASD group in comparison to the TDC group (Supplementary Figure S1).

**Figure 2:**
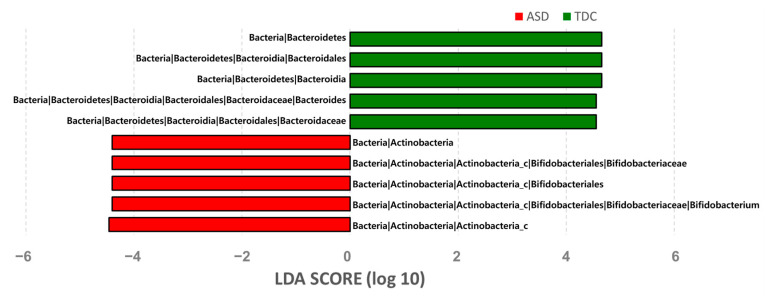
Spearman correlation between the relative bacterial abundances and Principal component 1 (PC 1) of weighted UniFrac analysis. LDA scores for discriminated bacterial taxa in the ASD and TDC.

LEfSe analysis further confirmed these significant differences. Remarkably, a significant increase in the relative abundances of Actinobacteria_p, Actinobacteria_c, Bifidobacteriales_o, *Bifidobacteriaceae*_f, and *Bifidobacterium*_g (at the phylum to genus) as well as a significant reduction in Bacteroidetes_p, Bacteroidia_c, Bacteroidales_o, *Bacteroidaceae*_f, and *Bacteroides*_g (at the phylum to genus) were observed in the ASD group as compared to the TDC group (LDA score > 4.0, *p* < 0.05) (Figure 2).

When we further identified correlations between SRS scores and *Bacteroides* spp. or *Bifidobacterium* spp. in a group of ASD (*n* = 54), only *Bacteroides* spp. had a weak correlation with total SRS score (rs = 0.28, *p* < 0.05, data not shown). However, there was no significant correlation between the two bacteria and the five subscales of SRS, including social awareness, social cognition, social communication, social motivation, and autistic mannerism. 

### 3.3. Microbial Diversitiy

**Figure 3:**
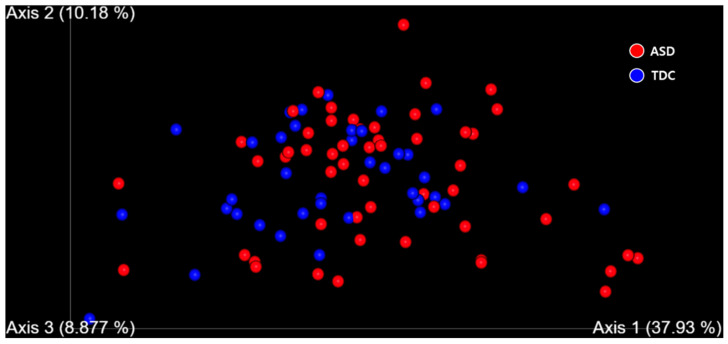
Beta diversity based on the weighted UniFrac distance in the ASD and TDC. Red and blue circles indicate ASD and TDC, respectively.

We analyzed the alpha diversity of ASD children and TDC based on richness, including Faith_pd and observed OTUs, and evenness, including the Shannon index and pielou_e. Alpha diversity analysis revealed no significant differences between ASD children and TDC, whereas beta diversity analysis based on the weighted UniFrac distances revealed that the bacterial community of ASD children was significantly different from that of TDC (PERMANOVA, pseudo-F = 2.649, *p* < 0.05) (Figure 3).

Moreover, principal component analysis was performed to determine which bacteria could effectively distinguish between the two groups. As a result, *Bifidobacterium* spp., which were abundantly present in the ASD group, including *B. catenulatum* and *B. longum*, were found to be the most influential factors that differentiated these two groups (Supplementary Figure S2).

### 3.4. Functional Analysis

**Figure 4:**
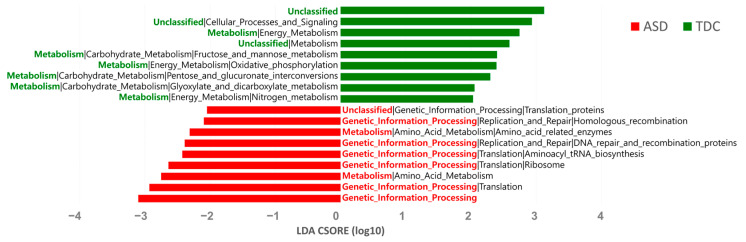
Predicted metagenome function based on KEGG pathways analysis.

The potential functions of the gut microbiota were predicted using PICRUSt analysis based on 16S rRNA sequences. Kyoto Encyclopedia of Genes and Genomes function analysis showed that genetic information processing and amino acid metabolism pathways were significantly higher in the ASD group than in the TDC group. In contrast, metabolism pathways, including carbohydrate and energy metabolism, were significantly higher in the TDC group (Figure 4).

### 3.5. Short-Chain Fatty Acid (SCFA) Analysis

**Figure 5:**
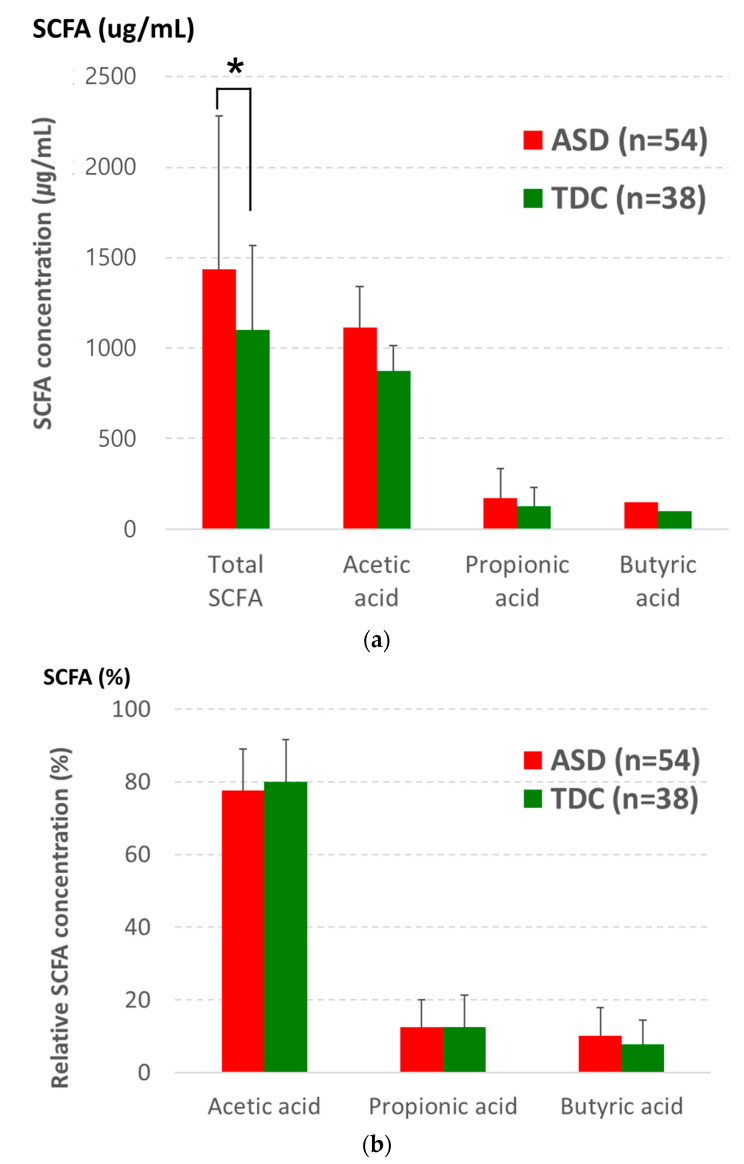
(**a**) Total SCFA and (**b**) the relative SCFA concentrations, including acetic acid and propionic acid butyric acid, in ASD and TDC. Error bars indicated standard deviation (SD). Asterisk indicates significance between groups (* *p* < 0.05).

The presence of major SCFAs, acetic acid, propionic acid, and butyric acid, was measured in both groups, finding that the total amount of SCFAs was significantly higher in ASD children (*p* < 0.05). However, acetic acid, propionic acid, and butyric acid levels did not show any significant differences between the two groups (Figure 5a). Furthermore, no differences were found between the two groups on comparing the relative concentrations of SCFAs (Figure 5b).

To explore the relationship between SCFA production and microbial richness, including Faith_pd and observed OTUs, we performed a correlation analysis between them within the ASD and TDC groups. We found that higher microbial richness in the TDC group was attributed to a higher butyric acid production rate. In contrast, microbial richness in the ASD group did not affect the butyric acid production rate (Table 2).

## 4. Discussion

In the present study, we characterized gut microbiota associated with ASD in Korean children. Using metagenomic analysis, we investigated the diversity of species in the samples. Among these, alpha diversity represents the richness and evenness of the microbiome in a single sample pool [45]. In the alpha diversity analysis of this study, we found no significant differences between the ASD and TDC groups, which was consistent with the results of several previous studies [21,46,47,48]. However, it should be noted that while other researchers have shown an increase in alpha diversity in the ASD group [49,50], some studies also showed a decrease in alpha diversity [27,51,52]. Beta diversity, on the other hand, refers to the dissimilarity between two microbial communities [45]. Differences between ASD and healthy control groups in beta diversity have been reported in previous studies, which was consistent with the current study’s results [21,27,49,52,53]. Through principal component analysis, we confirmed that *Bifidobacterium* spp., which was significantly higher in the ASD group than the TDC group in the taxonomic analysis, was most influential factor that differentiated these two groups.

To assess the composition of the gut microbiome in ASD, we conducted a microbial taxonomic analysis. As a result, we found a lower Bacteroidetes to Firmicutes ratio in the ASD group than in the TDC group due to a significant decrease in Bacteroidetes at the phylum level. A decreased Bacteroidetes to Firmicutes ratio in ASD patients has been consistently observed in previous studies [19,20,21] and has been associated with several inflammatory conditions, such as inflammatory bowel disease and obesity [54,55]. It is likely that the decreased proportion of Bacteroidetes in ASD may contribute to carbohydrate digestion and transport impairments. Williams et al., in particular, suggested that reduced intestinal gene expression involved in disaccharidase expression and hexose transporters found in ASD children was associated with microbial dysbiosis, possibly inducing GI disturbances, such as diarrhea and bloating [48].

Furthermore, we discovered that the relative abundance of the phylum Actinobacteria, including the genus *Bifidobacterium*, was significantly increased in the gut microbiota of the ASD group compared to that of the TDC group. *Bifidobacterium*, which is a common probiotic, promotes the production of different exopolysaccharides by acting as fermentable substrates for human gut bacteria [56]. However, in contrast to our findings, the proportion of *Bifidobacterium* has been reported to be reduced in ASD patients in previous studies [49,57]. Conversely, similar to our findings, Tomova et al. showed an increased *Bifidobacterium* abundance in ASD children, which was found to be decreased by probiotic supplementation. Liu et al. also demonstrated that vitamin A supplementation induced changes in *Bacteroidetes/Bacteroidales* as an ASD biomarker, consequently decreasing the proportion of *Bifidobacterium* [58].

In our study, the genus *Bacteroides*, belonging to the phylum Bacteroidetes, was found to be significantly decreased in ASD children as compared to that of TDC. *Bacteroides* species are one of the earliest colonizing and most numerically prominent microbial species in the gut microbiota [59]. Among them, *Bacteroides fragilis* is known to be effective in treating ASD-related symptoms. Hsiao et al., for one, confirmed that oral treatment using *Bacteroides fragilis* corrected gut permeability, altered microbial composition, and improved communicative, repetitive, sensorimotor, and anxiety-like behaviors in a maternal immune-activation mouse model [60]. Although we did not find statistically significant differences between the two, we also confirmed that the two species belonging to the *Bacteroides*, *Bacteroides vulgatus* and *Bacteroides dorei*, were decreased in the ASD group, similar to Hsiao et al.’s study.

Since there is a lack of consistency in reported ASD microbiome studies, it is difficult to use the gut microbiome composition as a predictive biomarker for ASD. Despite this, it has been repeatedly confirmed that the gut microbiome composition in ASD patients differs significantly from that of normal developing controls, and we provided further evidence for these differences in the present study. In particular, we confirmed the possibility that *Bacteroides* reduction could be an important feature in the gut microbiome profile of Korean ASD children, and we believe that it can be used therapeutically in the future. We also identified a significant correlation between *Bacteroides* spp. and total SRS score, which was reported to be correlated with severity of ASD [31]. Contrary to our expectation, *Bacteroides* spp. and SRS score showed a weak positive correlation, and there was no significant correlation between the two bacteria and the five subscales of SRS. These results also suggest that *Bacteroides* spp. may be just involved in the pathogenesis of ASD. We speculated that *Bacteroides* spp. might be not dose-dependently related to the severity level of ASD, but more complex factors are likely to be involved in the symptom severity. To determine the possible roles of the gut microbiota, we predicted the functional profiles of microbial communities using PICRUSt analysis. As a result, we found that several pathways, including genetic information processing and amino acid metabolism pathway, were significantly higher in the ASD group than in the TDC group. In a systemic review by Liu et al., a PICRUSt analysis was also conducted using raw data from previous studies [45]. Similar with our results, pathways, such as “ABC transporter”, “replication, recombination and repair proteins”, “lysine biosynthesis”, and “genetic information processing”, showed high discriminative power in ASD patients in Strati et al.’s study [21]. Moreover, in two studies by Kang et al., “metabolism” and “amino acid metabolism” pathways were found to be commonly increased in ASD patients [27,51]. Regarding the metabolic pathway, host metabolism has been found to be regulated by metabolites derived from the intestinal microbes [61]. For example, dysregulated metabolism of free amino acids (FAA) has been observed in children with autism [49]. Interestingly, glutamate, which acts as a neurotransmitter in the brain, is also one of these identified FAAs and has been implicated in ASD pathophysiology [62]. Furthermore, Ming et al. demonstrated abnormal amino acid metabolism in ASD children by showing decreased levels of amino acids, such as glycine, serine, and glutamyl [63]. Although these functional analysis results provide a key clue to understanding the underlying mechanisms of ASD pathogenesis, more studies are needed to definitively determine how the gut microbiota affects ASD pathogenesis.

SCFAs are metabolites produced by the bacterial fermentation of complex polysaccharides and resistant starches, such as cellulose and pectin. Notably, major SCFAs, such as acetic acid, propionic acid, and butyric acid, account for more than 90% of SCFAs and are produced and absorbed in the colon [64,65]. Recent studies have suggested that SCFAs play a role in microbiota–gut–brain communication; however, the role of SCFAs in ASD pathology remains controversial [66]. In this study, although we found an increased total amount of SCFAs in ASD children, we could not find any significant difference in SCFA levels between the two groups. These results were similar to a study conducted by Wang et al. showing elevated fecal total SCFA concentrations in children with ASD [67]. Additionally, animal studies have also confirmed that SCFAs induce ASD-like behaviors and brain alterations [68,69]. On the other hand, another study reported that the total amount of SCFAs decreased in ASD, which was partly associated with increased probiotic use [15]. They further explained that altered fecal SCFA levels in ASD can be influenced by several factors, such as gut microbiome composition, food intake, gut transit time variabilities, and gut permeability [15]. Therefore, more research is required in ASD patients to elucidate the role of SCFAs in ASD. When we analyzed the correlation between microbial richness and major three SCFAs, we found that the higher the microbial richness in the TDC group, the higher the butyric acid (BA) production, but there was no correlation between microbial diversity and BA production in the ASD group. In general, microbial diversity means a healthy gut environment; however, it seems that high microbial diversity does not simply mean increased SCFA, beneficial substance in gut environments, especially in pathological conditions such as ASD. The reason that the increase in microbial richness in the ASD group did not affect the SCFA level is probably because the intestinal environment related to ASD influenced the SCFA production. Despite the findings of this study, several limitations were noted. First, although we observed significant differences in the microbiome composition between the two groups at the phylum and genus levels, this was not observed at the species level. Second, in SCFA analysis, no significant differences were found in each subtype of SCFAs, acetic acid, propionic acid, and butyric acid. It can be inferred that one of the reasons behind this was not only due to the insufficient number of participants but also because ASD participants with various severities were included. Third, many ASD children who participated in this study did not receive treatment despite complaints of chronic constipation symptoms, resulting in the differences in physical hardness observed during fecal sample collection. Lastly, a variety of factors not considered in this analysis may have influenced the results. Therefore, it is necessary to interpret whether the differences in microbiome composition between ASD and TDC observed in this study is related to the pathogenesis of ASD or due to other factors, such as differences in nutritional habits. In fact, it has already been known that ASD children are generally picky eaters who do not prefer vegetable intake [70]. Therefore, it will be necessary to conduct studies with a large number of children in a more homogenous group in the future. If an analysis is included in consideration of various factors affecting the gut microbiome composition, such as the severity of GI and ASD symptoms, medications, and diet habits, more information will be obtained to understand the relationship between ASD pathogenesis and intestinal microbes.

Nevertheless, to the best of our knowledge, this is the first study to show that the gut microbiome and their metabolites in Korean ASD children were significantly different when compared with TDC. Given our findings, we suggested that *Bacteroides* reduction was one of the characteristics observed in children with ASD in Korea, and it is expected that this finding can be used to help and treat ASD in this population in the future.

## Figures and Tables

**Table 1:** Characteristics of ASD and TDC groups.

	ASD (N = 54), Mean ± SD	TDC (N = 38), Mean ± SD	*p*
Gender (*n*)			
Male	79.6% (43)	47.4% (18)	0.001
Female	20.4% (11)	52.6% (20)	
Age (year)	7.0 ± 2.1	6.0 ± 1.7	0.021
IQ	58.2 ± 17.8	103.0 ± 14.1	0.000
SRS (total, T score)	91.1± 14.5	43.0 ± 8.0	0.000
ADI-R (total)	31.7 ± 24.9	-	
ADOS-2 (total)	17.6 ± 15.4	-	

ASD, autism spectrum disorder; TDC, typical developing children; SD, standard deviation; IQ, intelligence quotient; SRS, Social Responsiveness Scale; ADI-R, Autism Diagnosis Interview-Revised; ADOS-2, Autism Diagnostic Observation Schedule-2.

**Table 2:** Spearman correlation analysis between SCFA and microbial richness. Spearman’s rank tests calculated the correlation coefficients between SCFA and microbial richness, including Faith_pd and observed OTUs. The results with significant correlation (*p* < 0.05) are presented as an asterisk mark.

Group		Total (N = 92)			ASD (N = 54)			TDC (N = 38)		
Relative SCFA		AA	PA	BA	AA	PA	BA	AA	PA	BA
Faith_pd	r_s_	−0.185	0.000	0.318	−0.006	−0.188	0.216	−0.428	0.264	0.490
	*p*	0.078	0.997	0.002 *	0.964	0.174	0.117	0.008 *	0.110	0.002 *
Observed OTUs	r_s_	−0.101	−0.011	0.253	0.083	−0.154	0.103	−0.319	0.169	0.438
	*p*	0.340	0.917	0.015 *	0.551	0.267	0.457	0.051	0.310	0.006 *

ASD, autism spectrum disorder; TDC, typical developing children; SCFA, short-chain fatty acid; AA, acetic acid; PA, propionic acid; BA, butyric acid. r_s_ indicates correlation coefficient, * *p* < 0.05.

## Data Availability

The raw Illumina read data for all samples have been deposited in the European Bioinformatics Institute European Nucleotide Archive database, under study accession number PRJEB45948.

## Supplementary Materials

The following are available online at https://www.mdpi.com/article/10.3390/nu13103300/s1, Figure S1: Microbial profiles at the at the species level, Figure S2: Spearman correlation between the relative bacterial abundances and Principal component 1 (PC 1) of weighted UniFrac analysis.
