# Supplementary material for: Altered Gut Microbiota in Korean Children with Autism Spectrum Disorders
Source: Nutrients. 2021 Sep 22;13(10):3300. doi: 10.3390/nu13103300 (PMC8539113; doi:10.3390/nu13103300)
Supplement: Supplementary file 1 [file nutrients-13-03300-s001.zip › nutrients-1392379-SI.pdf]

ASD	TDC	Wilcox. test	
1.593	0.372	0.799	Firmicutes|Negativicutes|Selenomonadales|Selenomonadaceae|Megamonas|Megamonas_funiformis
1.413	0.894	0.197	Firmicutes|Clostridia|Clostridiales|Lachnospiraceae|Eubacterium_g5|Eubacterium_hallii
0.960	1.785	0.412	Proteobacteria|Gammaproteobacteria|Enterobacteriales|Enterobacteriaceae|Escherichia|Shigella_flexneri
1.238	1.431	0.834	Bacteroidetes|Bacteroidia|Bacteroidales|Prevotellaceae|Prevotella|DQ796138_s
1.582	1.519	0.351	Bacteroidetes|Bacteroidia|Bacteroidales|Bacteroidaceae|Bacteroides|Bacteroides_xylanisolvens
1.507	1.746	0.131	Bacteroidetes|Bacteroidia|Bacteroidales|Rikenellaceae|Alistipes|Alistipes_onderdonkii
2.037	1.354	0.114	Firmicutes|Clostridia|Clostridiales|Lachnospiraceae|Fusicatenibacter|Fusicatenibacter_saccharivorans
1.690	2.089	0.743	Firmicutes|Clostridia|Clostridiales|Ruminococcaceae|Subdoligranulum|Gemmiger_formicilis
1.479	2.573	0.355	Bacteroidetes|Bacteroidia|Bacteroidales|Rikenellaceae|Alistipes|Alistipes_putredinis
2.833	1.650	0.525	Bacteroidetes|Bacteroidia|Bacteroidales|Bacteroidaceae|Bacteroides|Bacteroides_fragilis
2.479	2.192	0.095	Bacteroidetes|Bacteroidia|Bacteroidales|Bacteroidaceae|Bacteroides|Bacteroides_plebeius
2.712	2.094	0.331	Firmicutes|Clostridia|Clostridiales|Ruminococcaceae|Faecalibacterium|GG697149_s
2.736	2.298	0.381	Firmicutes|Clostridia|Clostridiales|Lachnospiraceae|Anaerostipes|Anaerostipes_hadrus
2.496	3.635	0.303	Bacteroidetes|Bacteroidia|Bacteroidales|Bacteroidaceae|Bacteroides|Bacteroides_ovatus
3.480	2.552	0.148	Actinobacteria|Actinobacteria_c|Bifidobacteriales|Bifidobacteriaceae|Bifidobacterium|Bifidobacterium_longum
3.164	3.366	0.769	Firmicutes|Clostridia|Clostridiales|Ruminococcaceae|Faecalibacterium|GL538271_s
2.866	4.153	0.930	Bacteroidetes|Bacteroidia|Bacteroidales|Bacteroidaceae|Bacteroides|Bacteroides_uniformis
2.393	5.358	0.076	Bacteroidetes|Bacteroidia|Bacteroidales|Bacteroidaceae|Bacteroides|Bacteroides_dorei
4.107	4.571	0.218	Firmicutes|Clostridia|Clostridiales|Ruminococcaceae|Faecalibacterium|FP929045_s
4.491	4.203	0.858	Firmicutes|Clostridia|Clostridiales|Lachnospiraceae|Blautia|Blautia_wexlerae
7.018	3.990	0.217	Actinobacteria|Actinobacteria_c|Bifidobacteriales|Bifidobacteriaceae|Bifidobacterium|Bifidobacterium_catenulatum
6.402	10.528	0.067	Bacteroidetes|Bacteroidia|Bacteroidales|Bacteroidaceae|Bacteroides|Bacteroides_vulgatus

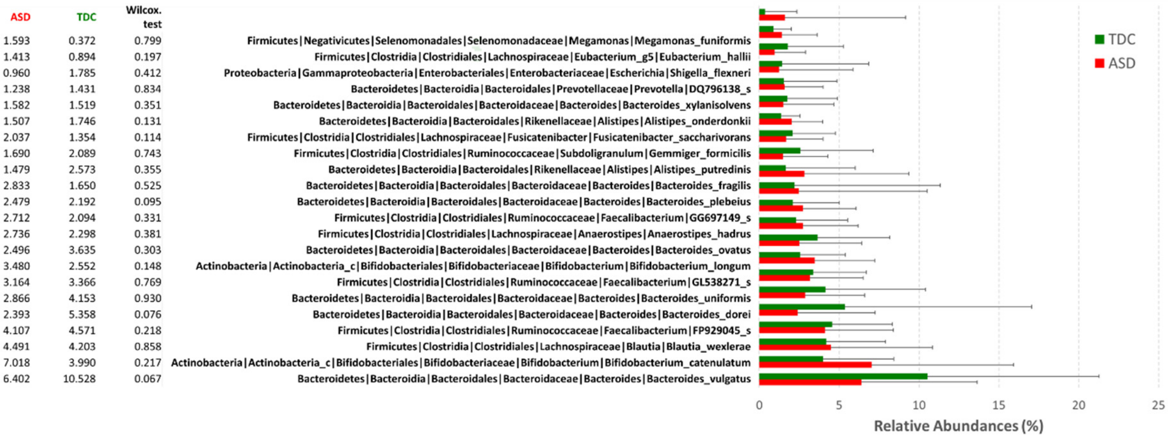

Supplementary Figure S1. Microbial profiles at the at the species level. The proportion of the bacterial composition was presented at a table. Error bars indicated standard deviation (S.D).

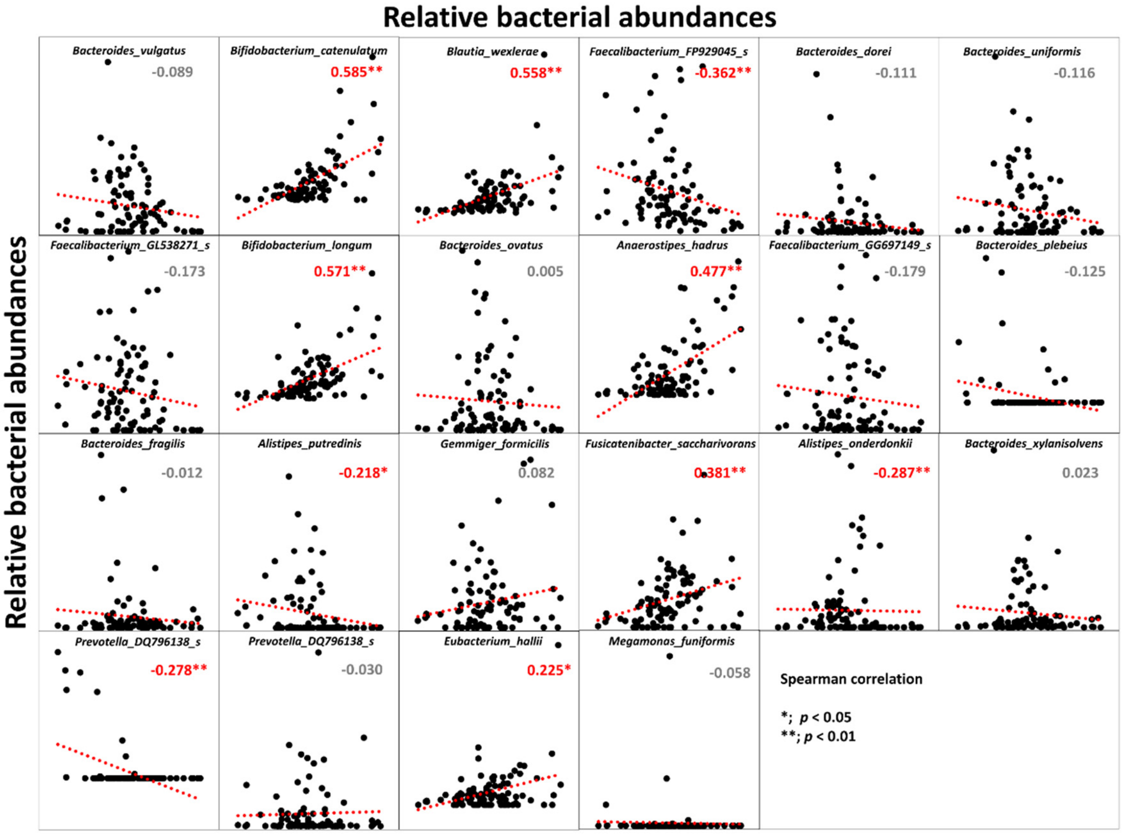

Supplementary Figure S2. Spearman correlation between the relative bacterial abundances and Principal component 1 (PC 1) of weighted UniFrac analysis. Asterisk indicates a significant correlation between relative bacterial abundances and PC 1 (*p<0.05, **p<0.01).
